# Losartan Potassium and Verapamil Hydrochloride Compound Transdermal Drug Delivery System: Formulation and Characterization

**DOI:** 10.3390/ijms232113051

**Published:** 2022-10-27

**Authors:** Yu-si Chen, Yi-yang Sun, Zi-chen Qin, Sai-ya Zhang, Wen-bo Chen, Yan-qiang Liu

**Affiliations:** College of Life Sciences, Nankai University, Tianjin 300071, China

**Keywords:** transdermal delivery, losartan potassium, verapamil hydrochloride, pharmacokinetics, skin irritation

## Abstract

In this study, we developed a sustained-release transdermal delivery system containing losartan potassium (LP) and verapamil hydrochloride (VPH). LP and VPH have low bioavailability and long half-life. Therefore, the development of an optimum administration mode is necessary to overcome these drawbacks and enhance the antihypertensive effect. A transdermal diffusion meter was used to determine the optimal formulation of LP-VPH transdermal drug delivery systems (TDDS). Based on in vitro results, a sustained-release patch was prepared. Physical characteristics, including quality, stickiness, and appearance, were evaluated in vitro, while pharmacokinetics and skin irritation were evaluated in vivo. The results showed that 8.3% polyvinyl alcohol, 74.7% polyvinylpyrrolidone K30, 12% oleic acid-azone, and 5% polyacrylic acid resin II provided an optimized TDDS product for effective administration of LP and VPH. Furthermore, in vitro and in vivo release tests showed that the system continuously released LP and VPH for 24 h. The pharmacokinetic results indicated that although the maximum concentration was lower, both the area under the curve from 0–time and the mean residence time of the prepared patch were significantly higher than those of the oral preparations. Furthermore, the prepared LP-VPH transdermal patch showed good stability and no skin irritation. The developed LP-VPH TDDS showed a sustained-release effect and good characteristics and pharmacokinetics; therefore, it is an ideal formulation.

## 1. Introduction

Hypertension (HT) is a chronic disease and common cardiovascular risk factor. It can be accompanied by complications and ranks third among the major causes of morbidity and mortality globally. HT affects approximately 1 billion people worldwide and is associated with approximately 58% of cases of hemorrhagic stroke, 50% of ischemic stroke, 55% of ischemic heart disease, and 58% of other cardiovascular diseases. Furthermore, the number of people affected by HT worldwide is expected to increase over the next decade [1,2]. The global prevalence of HT is currently > 31%. Moreover, from 2005 to 2015, the number of premature deaths increased to 10.7 million per year, suggesting that the burden of HT-related conditions has become a primary health challenge for society. It is estimated that the number of adults affected by HT globally will reach 1.56 billion by 2025 [3,4,5,6]. Therefore, it is important to develop effective therapies for HT [7].

Antihypertensive drugs, including angiotensin-converting enzyme inhibitors, angiotensin II receptor blockers (ARBs), calcium antagonists, β-blockers, and diuretics, are used to decrease blood pressure (BP) and play a role in alleviating the hypertensive complications mentioned above [8,9,10]. The initial recommendation for pharmacological treatment of HT is monotherapy and gradually increase the dosage of the selected drug until BP is under control [11,12]. However, increasing drug dosage can be associated with side effects, and most hypertensive patients require a combination of two or more antihypertensive drugs to control their BP (<140/90 mmHg). Therefore, combinatorial therapy is an ideal approach that may help obtain positive clinical outcomes while reducing the side effects associated with high drug doses [11,12,13,14].

Losartan potassium (LP), a non-peptide ARB, is usually administered orally at a dose of 50 mg/day. Verapamil hydrochloride (VPH), a calcium channel blocker, is administered orally at 40 mg once a day and can promote the antihypertensive efficacy of ARBs [15,16,17,18]. The combined use of ARBs and calcium channel blockers helps control BP. Furthermore, the combination is well tolerated, and significantly improves the efficacy and safety of these medications [18,19]. However, oral administration of LP and VPH results in an extensive and rapid first-pass effect, resulting in low bioavailability of 32% and 10–20%, respectively [16,20,21,22]. Therefore, optimum formulations are needed to enhance clinical outcomes. A transdermal drug delivery system (TDDS) may prevent pre-systemic metabolism and gastrointestinal degradation of these drugs and improve patient compliance, thereby solving the problems associated with traditional medical formulations [23,24]. Thus, an LP and VPH TDDS may help decrease BP more effectively.

To develop an optimal LP and VPH TDDS, we compared different types and concentrations of matrix patch skeletal materials and penetration enhancers, and different concentrations of pressure-sensitive adhesive (PSA), using in vitro skin permeation studies. The aim of this study was to develop an optimized TDSS, characterize its stability and adhesiveness, perform in vivo pharmacokinetic studies, and evaluate the sustained-release performance of LP and VPH from the selected patch.

## 2. Results

### 2.1. Preselection of Transdermal Systems

The results of the orthogonal experiments are listed in Table 1. We found that the final cumulative percentage of LP-VPH depended on the different components of the system. Among the skeletal materials, PVA and PVP K30 showed higher cumulative releases of 50.36% and 45.31%, respectively. An additional factor is the composition of the penetration enhancers. The cumulative drug release rate was highest when the ratio of OA to azone was 2:1, reaching 43.75%. Therefore, our preliminary matrix formulation was based on PVA as the primary material, PVP K30 as the secondary material, and OA:azone in a ratio of 2:1 as the penetration enhancer.

### 2.2. Effects of Skeleton Materials on the Transdermal Systems

**Figure 1:**
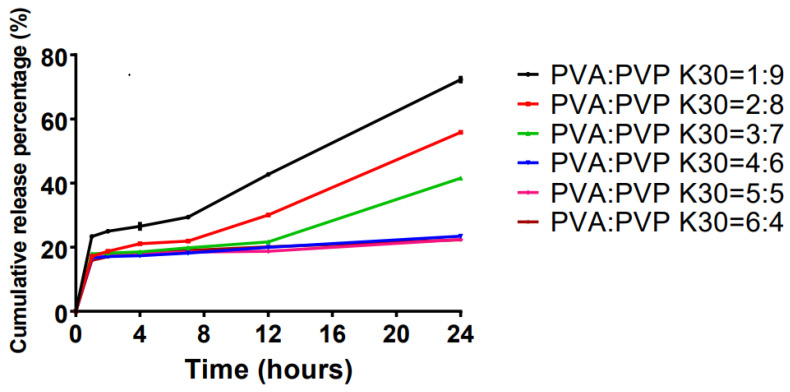
Effect of different ratios of PVA and PVP K30 on drug release in the LP-VPH compound transdermal drug delivery system (mean ± SD, n = 3).

After the preliminary matrix formulation was determined using orthogonal experiments, the optimal composition was evaluated by testing different proportions of PVA and PVP K30 (Table 2). As PVP K30 increased, the cumulative release of the LP-VPH increased significantly, as shown in Figure 1. Furthermore, when the ratio of PVA to PVP K30 was 1:9 (A1), the cumulative release percentage reached a maximum value of 72.30 ± 0.74%; therefore, this ratio of the skeleton materials was used in the transdermal system.

### 2.3. Effect of the Penetration Enhancer on the Transdermal Systems

**Figure 2:**
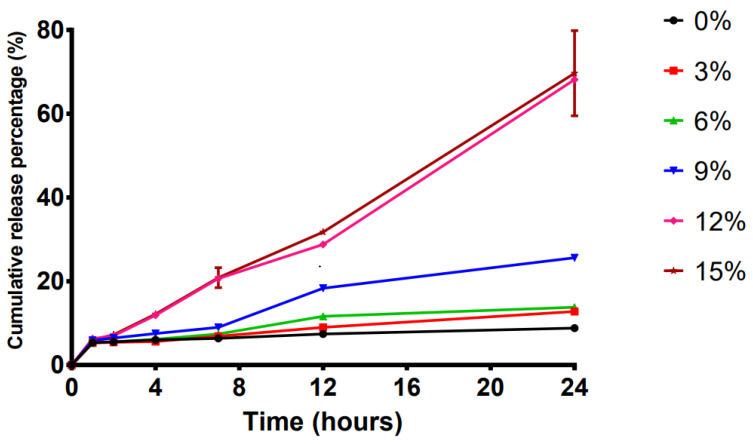
Effect of different concentrations of the oleic acid-azone compound on drug release in the LP-VPH compound transdermal drug delivery system (mean ± SD, n = 3).

To study the optimal penetration enhancers for the transdermal systems, six compositional variations (B1–B6) were selected (Table 2). The effect of different concentrations of a 2:1 mixture of OA:azone as the penetration enhancer on the release of the LP-VPH is presented in Figure 2. The drug release rate was observed to increase substantially with increasing concentration of the penetration enhancer. Samples B5 and B6 exhibited the highest drug release percentages of 68.17 ± 0.81% and 69.66 ± 10.16%, respectively, which was significantly higher than the value obtained with B1 (8.81 ± 0.13%, *p* < 0.05), with no significant difference observed between the release rates of B5 and B6. Therefore, we chose B5, a 12% OA-azone combination, for further use.

### 2.4. Effects of Polyacrylic Acid Resin II on the Transdermal Systems

**Figure 3:**
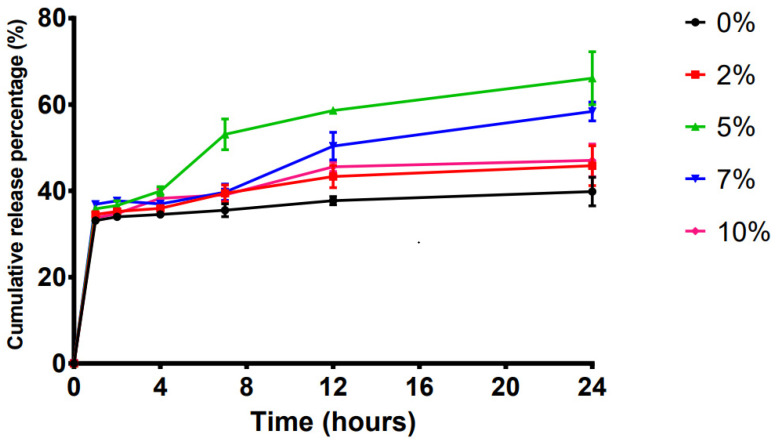
Effect of different concentrations of polyacrylic acid resin II on drug release in the LP-VPH compound transdermal drug delivery system (mean ± SD, n = 3).

A single-factor experiment was designed to study the optimal content of polyacrylic acid resin II as a pressure-sensitive adhesive (PSA) in the transdermal systems (Table 2). The effects of five different concentrations of polyacrylic acid resin II on the release of the LP-VPH with 5% polyacrylic acid resin II, demonstrating a peak cumulative release of 66.12 ± 6.15% (Figure 3). Therefore, 5% polyacrylic acid resin II was used in the transdermal system.

### 2.5. Scanning Electron Microscopy of TDDS

**Figure 4:**
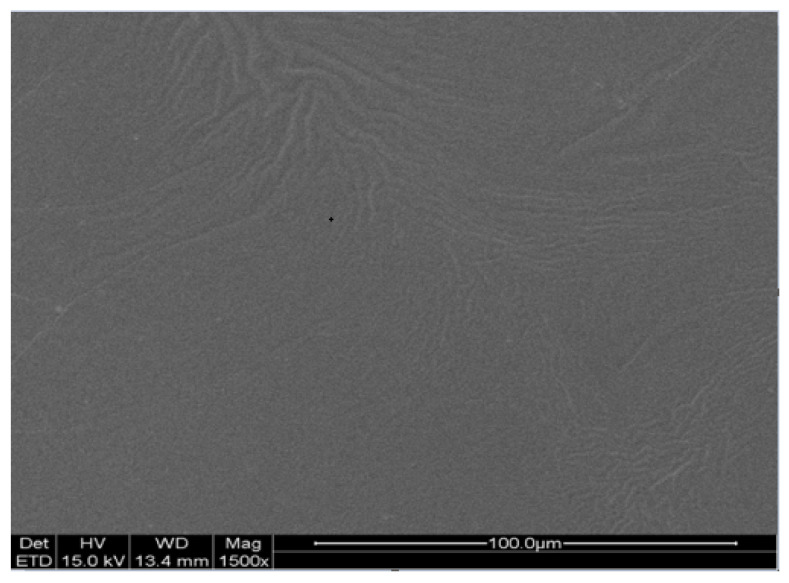
SEM images of the LP-VPH compound transdermal drug delivery system.

The scanning electron microscopy (SEM) image of the LP-VPH TDDS are shown in Figure 4. The image showed that the patch had good integrity and the drugs were evenly distributed in the polymer matrix, indicating that the prototype preparation had the necessary morphological characteristics for an effective TDDS.

### 2.6. Stability of TDDS

The quality control experiments showed that at high or low temperatures and under freeze-thaw conditions, there was no flow, wrinkling, or embrittlement of the patch surface. Additionally, no oil leakage was observed on the back of the patches, and no delamination was observed between the patches and the lining. These results indicate that there was no significant change in the appearance or structure of the TDDS. Furthermore, these results indicated that the patches had good stability.

### 2.7. Stickiness of the TDDS

To evaluate the stickiness of the TDDS, three main indicators—peel strength, shear strength, and hand adhesion—were selected. The results showed that the relative peeling time was 920 ± 21 s, while the shedding time was 1107 ± 85 s. Regarding hand adhesion, the displacement of the stainless-steel ball was 5.23 ± 0.49 cm. These results indicated that the LP and VPH sustained-release delivery systems had good adhesive qualities and met the requirements for use.

### 2.8. In Vivo Pharmacokinetic Studies for the TDDS

**Figure 5:**
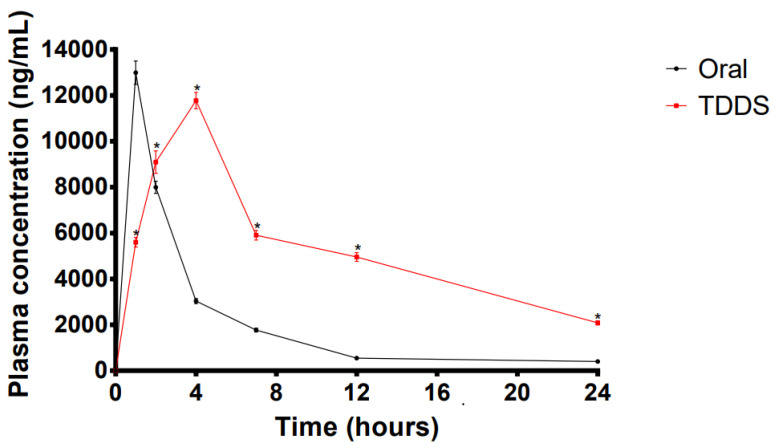
Plasma drug concentration-time curve analysis in rabbits after LP-VPH compound transdermal drug delivery system and oral administration (mean ± SD, n = 6, * *p* < 0.05, compared with the corresponding time points oral administration).

The pharmacokinetic performance of the optimized TDDS in rabbits was compared with that of orally administered LP-VPH. The plasma concentration-time profiles are shown in Figure 5 while the relevant pharmacokinetic parameters are summarized in Table 3 (the related values were calculated according to the DAS 2.0 procedure based on statistical moment theory). Compared to oral administration, the patches took longer to reach peak blood concentration, the C_max_ was relatively lower. Furthermore, the AUC_(0-t)_ and MRT of the patches were significantly increased (*p* < 0.0001), reaching 158175.20 ± 4206.84 ng·h^−1^·mL^−1^ (4-fold) and 4.63 ± 0.64 h (1-fold), respectively. In addition, the results summarized in Table 3 show that the ratio of AUC_(0-t)_ for the TDDS to that for oral administration (a result called the calculated relative bioavailability or F value) was 338.51%. Therefore, the developed LP-VPH TDDS showed significantly better sustained-release characteristics and higher bioavailability than those of oral administration.

### 2.9. Skin Irritation Tests

**Figure 6:**
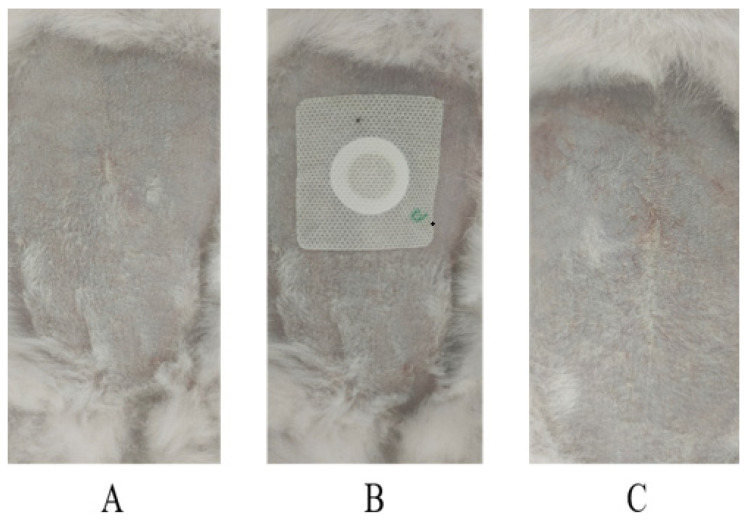
Skin irritation study for the LP-VPH compound transdermal drug delivery system showing (**A**) before application of the patch, (**B**) during the application of the optimized patch, and (**C**) after removal of the patch (n = 6).

The results of the skin irritation tests following the use of the LP-VPH TDDS are shown in Figure 6 and Table 4. The results showed that the patches did not induce erythema or edema in the rabbit skin. The erythema and edema irritation scores were <0.5. Furthermore, there was no significant difference between the use of blank TDDS or LP-VPH TDDS in terms of skin irritation. Therefore, LP-VPH TDDS are safe and had no irritating effects on the skin.

## 3. Discussion

The TDDS is easy to use, controllable, and can be self-administered by patients in a range of different circumstances [25]. Among the available types of TDDS, patches are the most used because of their uncomplicated design, convenient application, and low production cost. Patches are classified as either reservoir or matrix types based on their design characteristics [26]. Drugs loaded into a reservoir-type patch can easily leak if the patch is cut or accidentally damaged, whereas the use of a matrix patch design can overcome this problem [27]. Therefore, we selected a matrix-type TDDS design to formulate LP-VPH sustained-release delivery systems.

Matrix materials are important components of patches. HPMC, which is available at different viscosity grades, is non-toxic and has fast gel formation and compressibility characteristics. Therefore, HPMC is convenient and widely used in the preparation of pharmaceutical products [28,29]. HC is a non-ionic and non-toxic carbohydrate polymer used as a viscosity enhancer [30,31]. CMC-Na is an anionic and water-soluble polymer with high water solubility and hydrophilicity, which has resulted in its use in pharmaceutical preparations [32]. EC is an ideal polymer for various purposes and can prolong drug release, improving patient compliance [29]. PVP K30 is a non-ionic polymer that has been widely used in medicine. Moreover, PVP K30 has been shown to maintain a relatively long drug release time [33,34]. It is a water-soluble polymer with good adhesion, flexibility, and film-forming properties, and is suitable for use in various medical applications [32]. The physicochemical properties and bioavailability of drugs can be improved when polymers are used in combination compared with their use in single preparations [35]. Therefore, in this study, we tested two combinations of polymers as optimal matrix skeleton materials. The orthogonal experimental results showed that the combination of PVA and PVP K30 had a high cumulative permeability for LP-VPH. Therefore, these polymers were used as primary and secondary matrix skeleton materials. The release rates of LP-VPH were positively correlated with the amount of PVP used, indicating that a PVA/PVP K30 ratio of 1:9 was the optimal matrix material composition.

Owing to the barrier characteristics of the skin, TDDS are unable to achieve complete penetration. Therefore, are several approaches were developed to reduce the skin barrier resistance, including the use of chemical enhancers, microneedles, and lasers [36]. We used two chemical penetration enhancers: OA and azone. OA can extract endogenous lipids from the skin to disrupt highly compact intercellular lipid accumulation and produce large aqueous pores in the stratum corneum to promote transdermal drug diffusion [37]. Azone can infiltrate the lipid bilayer between epidermal cells, changing the well-organized tissue, increasing the lipid fluidity of the stratum corneum, and boosting the drug permeation coefficient [38,39]. We evaluated experimental formulations treated with OA, azone, and the combination of the two in mice and found that the use of 12% OA: azone in a 2:1 ratio as the enhancer system induced a relatively high release of LP-VPH. Thus, these are considered optimal penetration enhancers.

PSA is a viscoelastic material that can provide firm bonding to the skin surface when a slight external pressure is applied, and it exerts a strong retention force, leaving no residue after removal from the surface [40]. Furthermore, PSA guarantees good drug permeability, indicating that PSA is vital for patch formulation [41]. Polyacrylic acid resin II can adhere to biological tissues and shows excellent sustained-release efficiency in the skin [42]. Our results demonstrated that including 5% polyacrylic acid resin II maintained the developed patch moderately adhesive qualities and optimized the cumulative release of the LP-VPH from the sustained-release TDDS.

Integrity, stability, and stickiness are vital quality metrics for TDDS. Therefore, we analyzed the characteristics of our LP-VPH TDDS. The SEM images showed that the prepared patches possessed the appropriate shape characteristics. The results of the stability experiments revealed no significant changes in the appearance of the prepared patches. Furthermore, the peel strength, hand adhesion, and shear strength experiments demonstrated that the prepared patches exhibited excellent adhesive performance. In summary, our optimized sustained-release TDDS for LP and VPH was structurally intact, stable, and adhered to the skin with no irritation.

Pharmacokinetic analysis showed that the AUC_(0-t)_ and MRT values obtained using the patches were significantly greater than those obtained following oral administration. Furthermore, plasma concentrations of LP and VPH remained above 2 μg/mL for 24 h, which is an effective therapeutic concentration. The AUC_(0-t)_ ratio comparing the LP-VPH TDDS and oral administration was analyzed. The bioavailability was notably augmented, reaching 338.51%, indicating that changing the dosage form of the drug overcame the issue of the first-pass effect observed with oral administration.

We also evaluated whether our TDDS produced skin reactions such as erythema and edema [43,44]. No erythema or edema was observed on the skin of the rabbits, indicating that our LP-VPH TDDS did not cause skin damage.

## 4. Materials and Methods

### 4.1. Materials

VPH (V820460) and ethyl cellulose EC (E809017, 18–22 mPa·s) were purchased from Shanghai Macklin Biochemical Technology Co., Ltd. (Shanghai, China). LP (S60909), carboxymethyl cellulose sodium (CMC-Na, S14016, 300–800 mPa·s), hydroxypropyl methylcellulose (HPMC, S14174, 4000–6500 mPa·s, HPMC K_4m_; 30,000 mPa·s, HPMC K_30m_; 100,000 mPa·s, HPMC K_100m_), polyacrylic acid resin II (S30569), and hydroxyethyl cellulose (HC, YY11927, 38,000–42,000 mPa·s) were purchased from Shanghai Yuan-ye Biotechnology Co., Ltd. (Shanghai, China). Potassium dihydrogen phosphate was purchased from Tianjin Huihang Chemical Technology Co., Ltd. (Tianjin, China). Azone, oleic acid (OA), polyvinylpyrrolidone (PVP K30, C20201, 3.4 mPa·s), and polyvinyl alcohol (PVA, D08019, 44.0–54.0 mPa·s) were purchased from Tianjin Damao Chemical Reagent Co., Ltd. (Tianjin, China). Methanol, ethanol, and acetonitrile were purchased from Tianjin Concord Technology Co., Ltd. (Tianjin, China). Methanol and acetonitrile were high-performance liquid chromatography (HPLC) grade, whereas the other reagents were of analytical grade.

### 4.2. Animals

Male Kunming mice (6 weeks old, weighing 16–18 g) and male Chinese rabbits (6 months old, weighing 2.0 kg) were purchased from Beijing SPF Biotechnology Co., Ltd. (Beijing, China). The mice were housed under standard laboratory conditions (12 h light/dark cycle, ~25 °C) and ad libitum food and water. The animals were housed in individual cages and fed a standard diet. Animal care and experimental protocols follow institutional guidelines for the health and care of experimental animals. The study protocol was approved by the Committee for Ethics of Animal Experiments at Nankai University, Tianjin, China (No. 16/06042018).

### 4.3. Drug Evaluation Conditions

The LP and VPH concentrations in the samples were evaluated using a CoM 6000 HPLC system (Albany, New York, NY, USA) with a Comatex C18-AB column (250 × 4.6 mm, 5 µm; 115 Corp, Plainfield, IL, USA) at 254 nm. The mobile phase consisted of 0.02 mol/L potassium dihydrogen phosphate and acetonitrile (60:40, *v*/*v*), at a flow rate of 1.2 mL/min at 25 °C. All sample solutions were degassed and the injection volume was maintained at 20 µL.

### 4.4. Preparation of Mice Abdominal Skin

Cervical dislocation was induced in Kunming mice under anesthesia using 4% chloral hydrate. The hair of the mice was removed using a hair removal device, and a sample of skin (approximately 2 cm × 2 cm) was cut from the abdominal region. After removing the subcutaneous adipose tissue and adhesive layer, the skin sample was repeatedly washed with normal saline and stored at −20 °C in a petri dish covered with filter paper wetted with normal saline. Skin samples were thawed at 25 °C before use.

### 4.5. Optimization of Transdermal Systems

An orthogonal design using an L_25_ (5^5^) array was established in in vitro transdermal experiments and used to select matrix types for transdermal systems. The choice of the first and second skeletal materials and penetration enhancers were the three factors in the orthogonal array (Table 5). Furthermore, different variations in each factor were taken as the levels in the orthogonal array. Transdermal systems were prepared using different ratios of PVA:PVP K30, penetration enhancers, and polyacrylic acid resin II (Table 2) to determine the optimal formulation.

### 4.6. In Vitro Release Studies

Drug release studies were conducted using a Franz transdermal diffusion meter (TP-6 transdermal diffusion meter; Tianjin Jingtuo Instrument Technology Co., Ltd., Tianjin, China). The excised skin was flattened and firmly affixed between the donor and receptor compartments. Each receptor compartment was filled with 15 mL of phosphate-buffered saline (PBS) at pH 6.8, maintained at 37 ± 0.5 °C using a thermostat, and stirred with a magnetic bar at 70× *g* to maintain a uniform distribution. Sample aliquots of 0.5 mL were extracted from the receptor compartment at different time intervals (1, 2, 4, 7, 12, and 24 h) and replaced by an equal volume of PBS (pH 6.8). The samples were filtered using a 0.22 µm pinhole filter (Scienhome Scientific Technology Development Co., Ltd., Tianjin, China) before injection into the HPLC (refer to Section 4.3, and the concentration of the drug was calculated based on the established standard curve).

### 4.7. Preparation of TDDS

The drug-containing transdermal systems were prepared using a solvent evaporation technique, according to the ratios described in Table 6, using 50 mg LP and 40 mg VPH. Each component was dissolved in 75% ethanol and magnetically stirred (60 °C, 30× *g*) for 2 h. After the solution was evenly mixed, it was degassed by ultrasonication at room temperature for 30 min. Next, the mixture was spread on an anti-adhesion layer and dried at 45 °C for 15 min, and subsequently pressed onto the dialysis membrane surface to obtain the transdermal systems. The patches were stored at room temperature in sealed containers.

### 4.8. Scanning Electron Microscopy Analysis

The external morphology of the TDDS was analyzed using a scanning electron microscope (QUANTA 200, FEI Company, Hillsboro, OR, USA).

### 4.9. Quality of Prepared TDDS

#### 4.9.1. High-Temperature Experiment

The LP-VPH TDDS were placed in an oven at 50 ± 5 °C for seven days. The exterior appearance and spreading properties of the patches were examined. Experiments were carried out in triplicate.

#### 4.9.2. Cold Resistance Experiment

The LP-VPH TDDS were placed in a refrigerator at 2–8 °C for 7 days. This process was repeated in triplicate, and the exterior appearance and ductility of the patches were examined at room temperature.

#### 4.9.3. Freeze-Thaw Experiment

The LP-VPH TDDS were placed alternately in a freezer at −20 °C and in an oven at 37 ± 0.5 °C for 8 days. This process was repeated in triplicate, and the exterior of each patch was examined, as described above.

### 4.10. Stickiness of the Prepared TDDS

#### 4.10.1. Peel Strength Experiment

The LP-VPH TDDS were attached to a vertically placed clean stainless-steel plate and smoothed down to ensure that there were no bubbles at the bonding site. After 30 min, the upper end of each patch was folded in half at 180°, and a 150 g steel ball weight was hung to strip the patch by gravity. This process was carried out in triplicate, and the time required for complete stripping of the patches was recorded. Peel strength was determined based on the time at which the patches fell from the apparatus.

#### 4.10.2. Shear Strength Experiment

Each LP-VPH transdermal patch was tightly attached to a vertically placed, clean stainless-steel plate, and a 150 g steel ball weight was hung from its lower end after 30 min, in a direction parallel to gravity. This process was repeated three times, and the time taken for the patch to be completely removed from the plate was recorded. Shear strength was determined based on the time at which the patches fell from the apparatus.

#### 4.10.3. Hand Adhesion Experiment

LP-VPH TDDS were laid flat on a table and a stainless-steel ball with a 5 cm diameter was rolled down a panel inclined at an angle of 25° over the patches. The process was repeated three times, and the distance that the steel ball passed through the adhesive surface when placed horizontally was determined as the hand adhesion force.

#### 4.10.4. In Vivo Pharmacokinetic Studies

Rabbits were assigned to three groups: A, B, or C. The abdominal hair of rabbits in groups B and C was shaved off with an electric hair remover, and after cleaning the skin with normal saline, the optimized TDDS were applied to the abdomen of group B and the blank patches were applied to the abdomen of group C, as shown in Figure 6. Group A was administered an oral gavage. The oral procedure was to fix the rabbits with an animal fixator and place a special mouth expander into their mouth attached with a rope. An elastic rubber catheter was inserted through a small round hole in the mouth expander and pushed into the esophagus along the posterior pharyngeal wall. A solution consistent with that of the TDDS drug concentrations was then added. After gastric perfusion, the catheter was pulled out and the mouth opener was removed. Next, 1 mL aliquots of blood were collected from the marginal auricular vein of the experimental animals at different time intervals (1, 2, 4, 7, 12, and 24 h), placed in heparinized tubes, centrifuged at 1500× *g* for 10 min, and the plasma supernatant was stored at −70 °C until further analysis. HPLC analysis was carried out by transferring 200 μL of the supernatant to a 1.5 mL polyethylene centrifuge tube. The tubes were then mixed with 300 μL acetonitrile, centrifuged at 2500× *g* for 10 min, and filtered through a 0.22 µm membrane.

#### 4.10.5. Skin Irritation Studies

After patch treatment in groups B and C, the test substances were removed from groups B and C simultaneously. Erythema and edema in the depilated part of the abdomen of the two rabbit groups were observed and recorded at 0 and 72 h after removing the test substance. Erythema and edema of the application sites were scored daily, as shown in Table 7.

### 4.11. Data Analysis

The pharmacokinetic parameters of the conventional oral and sustained-release transdermal preparations were analyzed, including the maximum plasma concentration of the drug (C_max_), time to reach C_max_ (T_max_), mean residence time (MRT), and area under the plasma concentration-time curve from time 0 to t (AUC_(0-t)_). Two-tailed *t*-tests were performed for C_max_ and AUC_(0-t)_ analyses, and a non-parametric test was performed for T_max_ analysis. AUC_(0-t)_ was calculated using the trapezoidal rule.

All results are represented as the mean ± standard deviation (SD), and comparisons of more than two groups were performed using one-way analysis of variance (ANOVA), and comparisons of two groups were performed using Student’s *t*-test. Statistical significance was set at *p* < 0.05.

## 5. Conclusions

Using in vitro and in vivo data, we prepared an LP-VPH TDDS and optimized a formulation containing PVA, PVP K30, OA, azone, and polyacrylic acid resin II. We observed that the patches had good integrity without any cracks and ideal stability, adhesion, and skin applicability, without the development of irritation. Moreover, the patches slowly released LP and VPH, and prolonged their antihypertensive effects. However, further clinical studies are required to fully evaluate its prospects.

## Figures and Tables

**Table 1:** L_25_ (5^5^) orthogonal experiments.

	First Skeleton Materials (a)	Second Skeleton Materials (b)	Penetration Enhancers (c)	Cumulative Release Rate (%)
1	1	1	1	19.24
2	1	2	2	23.99
3	1	3	3	24.51
4	1	4	4	26.09
5	1	5	5	28.00
6	2	1	2	41.24
7	2	2	3	40.65
8	2	3	4	28.54
9	2	4	5	44.44
10	2	5	1	40.31
11	3	1	3	41.23
12	3	2	4	38.09
13	3	3	5	42.47
14	3	4	1	41.11
15	3	5	2	33.20
16	4	1	4	47.90
17	4	2	5	41.56
18	4	3	1	42.85
19	4	4	2	36.77
20	4	5	3	36.06
21	5	1	5	41.85
22	5	2	1	32.42
23	5	3	2	52.93
24	5	4	4	78.13
25	5	5	3	46.63
K1	121.83	191.44	175.93	
K2	195.18	176.71	188.13	
K3	196.10	191.31	189.08	
K4	205.14	226.53	218.74	
K5	251.95	184.20	198.31	
k1	24.37	38.29	35.19	
k2	39.04	35.34	37.63	
k3	39.22	38.26	37.82	
k4	41.03	45.31	43.75	
k5	50.39	36.84	39.66	

**Table 2:** Formulation of LP-VPH compound transdermal drug delivery system.

Formulation	PVA:PVP K30	OA:azone = 2:1 (%, *v*/*v*)	Polyacrylic Acid Resin II (%, *v*/*v*)
A1	1:9		
A2	2:8		
A3	3:7		
A4	4:6		
A5	5:5		
A6	6:4		
B1		0	
B2		3	
B3		6	
B4		9	
B5		12	
B6		15	
C1			0
C2			2
C3			5
C4			7
C5			10

**Table 3:** Pharmacokinetic parameters (n = 6).

Parameters	Oral Administration	LP-VPH Compound Patch
C_max_ (ng/mL)	12,993.02 ± 511.01	11,774.14 ± 360.14
T_max_ (h)	1	4
AUC_(0-t)_ (ng·h^−1^·mL^−^^1^)	46,726.65 ± 821.11	158,175.20 ± 4206.84 ***
MRT (h)	3.39 ± 0.04	4.63 ± 0.64
F (%)	-	338.51

C_max_: peak concentration; AUC: area under drug time curve; MRT: mean residence time; F (%): the calculated relative bioavailability is AUC_(0-t)_ for the TDDS/AUC_(0-t)_ for the oral administration; *** *p* < 0.0001, compared with oral administration.

**Table 4:** Results of skin irritation (n = 6).

Administration	Erythema		Edema	
	0 h	72 h	0 h	72 h
Blank patch	0.00 ± 0.00	0.24 ± 0.09	0.00 ± 0.00	0.17 ± 0.05
Compound patch	0.00 ± 0.00	0.27 ± 0.11	0.00 ± 0.00	0.19 ± 0.05

**Table 5:** Orthogonal test table L_25_ (5^5^).

Option		Factors		
		First Skeleton Materials (a)	Second Skeleton Material (b)	Penetration Enhancers (c)
Level	1	HPMC K_4M_	HPMC K_100M_	OA
	2	HPMC K_30M_	CMC-Na	azone
	3	HC	EC	OA:azone = 1:1
	4	PVP K30	PVP K30	OA:azone = 2:1
	5	PVA	PVA	OA:azone = 1:2

**Table 6:** LP-VPH compound transdermal drug delivery system optimized formulation.

Components	Usage (%)
PVA	8.3% (*w*/*w*)
PVP K30	74.7% (*w*/*w*)
OA:azone = 2:1	12% (*v*/*v*)
polyacrylic acid resin II	5% (*w*/*w*)

**Table 7:** Scoring criteria for skin irritation.

Symptoms	Skin Condition	Evaluation Score
erythema	None	0
	Very slight (Almost observable)	1
	Well defined	2
	Moderate to severe	3
	Severe to slight eschar formation	4
edema	None	0
	Very slight (Almost observable)	1
	Slight (Obvious uplift at the edge of the area)	2
	Moderate (Area edge humps 1 mm)	3
	Severe (Area edge humps > 1 mm)	4

## Data Availability

The study did not report any data.
